# Blood shear stress during the cardiac cycle and endothelial cell orientation and polarity in the carotid artery of male and female mice

**DOI:** 10.3389/fphys.2024.1386151

**Published:** 2024-07-12

**Authors:** Nabil Nicolas, Alexandre de Tilly, Etienne Roux

**Affiliations:** ^1^ Biologie des Maladies Cardiovasculaires, INSERM, U1034, University of Bordeaux, Pessac, France; ^2^ Hemovis, Fontenay-sous-Bois, France

**Keywords:** ultrasound imaging, blood viscosity, Fahreaeus-Lindqvist-effect, carotid artery, sex effect

## Abstract

**Introduction:** Blood flow produces fluid shear stress (SS), a frictional force parallel to the blood flow, on the endothelial cell (EC) layer of the lumen of the vessels. ECs themselves are sensitive to this frictional force in terms of directionality and intensity. The aim of this study was to determine the physiological shear stress value during the cardiac cycle and EC polarity and orientation from blood flow in healthy male and female mouse carotid artery.

**Methods:** Experimentation is done on anesthetized male and female 8-week-old C5BL/6J mice. *In vivo* measurements of maximum blood velocity and vessel diameter in diastole and systole were performed on the right common carotid artery by Doppler ultrasound imaging. Blood viscosity (total and plasmatic) and hematocrit were determined on blood samples. For SS calculation, we developed a new method assuming heterogenous blood flow, i.e., a red cell central plug flow surrounded by a peripheral plasma sheath flow, and computing SS from vessel diameter and hemodynamical measurements (maximal blood velocity, hematocrit and plasmatic viscosity).

**Results:** Results were compared with the classical method assuming a homogenous blood flow with constant apparent total blood viscosity. EC polarity and orientation were determined *ex vivo* on the carotid endothelium by confocal imaging after labeling of the EC nucleus and Golgi apparatus. Diastolic and systolic SS were 6 ± 2.5 Pa and 30 ± 6.5 Pa, respectively. Total blood and plasmatic viscosity was 4 ± 0.5 cP and 1.27 cP, respectively. ECs were polarized and significantly oriented against blood flow. No sex difference was identified.

## Introduction

Blood flow produces fluid shear stress, a frictional force parallel to the blood flow, on the endothelial cell (EC) layer of the walls of the vessels, the wall shear stress (WSS). Due to hemodynamic variations during the cardiac cycle, the WSS is not a constant but varies between diastole and systole. ECs themselves are sensitive to this frictional force in terms of directionality and intensity, inducing local short-term (vasomotricity) and long-term (remodeling) alteration of the vessel, which in turn modifies the hemodynamical characteristics of the blood flow (Baeyens et al., 2016). It has been shown that this feedback loop contributes to the maintenance of the shear stress value in a narrow physiological range, the so-called WSS homeostasis, in which EC planar polarity plays a key role. Theoretical and experimental investigation have shown that this WSS homeostasis contributes the functional efficiency of the vascular network, and loss of WSS homeostasis robustness or homeostasis disruption is involved in vascular impairment and disease (Ong et al., 2020; Roux et al., 2020). A large number of publications have been dedicated to the investigation of the effect of WSS on ECs and vessel morphogenesis and remodeling (McCue et al., 2006; Chen and Tzima, 2009; Poduri et al., 2017; John et al., 2018; Lee et al., 2022). However, the availability in the literature of WSS physiological values is limited, in mice, an animal model largely used in biomedical research. The aim of this study is to determine *in vivo* physiological WSS value during the cardiac cycle in healthy male and female mouse carotid artery by *in vivo* measurement of blood flow velocity and vessel diameter combined with blood viscosity determination, and EC polarity and orientation from blood flow.

For a given fluid, the shear stress depends on the viscosity of the fluid and the shear rate it generates. On this basic principle, several methods exist for the calculation of the WSS, both *in vivo* and *in vitro*, depending, on the one hand, on the parameters that can be experimentally determined and, on the other hand, on theoretical hemodynamic assumptions about flood flow grounding the mathematical formulation of WSS calculation. Classically, calculation of WSS is based on the assumption that blood can be considered as a “Newtonian” fluid, i.e., a homogenous fluid with constant viscosity, following the Poiseuille’s law (Roux et al., 2020). However, it is known, since the primary work of Fahraeus and Lindqvist, that in narrow vessels (diameter inferior to 0.3 mm) the hemodynamic flow follows a structure of a plug flow of the red cells surrounded by a sheath flow of plasma, the so-called “Fahraeus-Lindqvist” effect (Fåhraeus, 1929; Fåhræus and Lindqvist, 1931; Barbee and Cokelet, 1971). Fahraeus and Lindqvist showed that in such narrow vessels the Poiseuille’s law, relative to whole blood viscosity, is not valid. In this case, the WSS depends on the plasma layer, i.e., plasma thickness and viscosity, and on the suspension state of the red cells, i.e., the plug flow thickness, and the vessel radius. Though the Fahraeus-Lindqvist effect occurs in narrow vessels, it has been evidenced that “Fahraeus-type” heterogenous blood behavior, i.e., with a red cell central plug flow surrounded by a peripheral plasma sheath flow, actually occurs for vessel diameter superior to 0.3 mm (Aarts et al., 1984; Aarts et al., 1988; Yeleswarapu et al., 1998; Hoskins, 2011; Secomb and Pries, 2013). Additionally, recent work of Thurston and Tilly showed that such a central plug/peripheral plasma sheath effect appears well in vessel-like geometries, namely, glass and polydimethylsiloxane capillaries, allowing the development of a new device for the measurement of total blood and plasmatic viscosities (de Tilly et al., 2023). One specific objective of this study was hence to propose a method based on the blood plug and sheath flow separation principle that grounded both the mathematical formulation of the shear stress exerted on the vessel wall and the obtention of experimental data for blood viscosity, compared to the classical one assuming Poiseuille’s law Newtonian fluid properties of the blood.

## Materials and methods

### Animal preparation

Experiments were done on male and female 8-week-old C5BL/6J mice, in accordance with National and European Union guidelines for experimental animal use, using procedures approved by the local Ethical Committee and authorized by National institution [#32550-2021100615085247 v4]. Food and water were available *ad libitum*, with a 12 h dark/light cycle. Mice were anaesthetized by gas (Vetflurane^®^ 1,000 mg/g, Virbac). They were placed on their backs and their legs taped to a 38°C heating platform. Thoracic hair was removed using a depilatory cream (Veet MinimaTM Sensitive Skin Depilatory Cream, Reckitt, Slough, United States). An ultrasound gel (neojelly^®^us, Asept InMed^®^, Quint-Conserves, France) was applied to the thoracic area. After ultrasound recording, an 850 µL blood sample was taken intravenously via the retro-orbital route using a piece of capillary pipette (ringcaps^®^50µL, HIRSCHMANN, Eberstadt, Germany) in an EDTA tube (4 mL EDTA K2 (7.2 mg) Tube BD^®^ Vacutainer^®^, Becton, Dickinson and Company, United States). Mice were then euthanized without recovery by intraperitoneal injection, using a 25G needle, of 300 µL of sodium pentobarbital (Exagon^®^ à 400 mg/mL, Axience, Pantin, France) diluted in physiological solution.

### *In vivo* measurement of carotid diameter and blow flow velocity

After thoracic depilation, *in vivo* measurement of blood flow and vessel diameter of the right common carotid artery are performed on anesthetized mice by Doppler ultrasound imaging, using a Vevo 2,100 Imaging Platform ultrasound scanner (VisualSonics, Toronto, Canada).

The diameter of the right common carotid artery was measured on series of images of the artery acquired in B mode with a gain of 27 dB, an image frequency of 78, a depth of 13 mm, a width of 10.36 mm, and a focal zone framing the carotid artery. The diameter was measured (in mm) in diastole and systole over the same cycle. Measurements were repeated on three different cycles, located at a distance from the inspiration, and the average of the three was automatically calculated by the device.

The velocity of the circulating blood was measured in the middle of the right common carotid artery between the first rib and the bifurcation between the right internal and external carotid arteries, in the center of the carotid artery to obtain the maximum blood velocity. Maximum blood velocity was recorded as a function of time in Pulse Wave mode with a gain of 30 dB, a beam angle of 0° and a 1,000 Hz filter, and with a flow measurement zone located at a depth of 8 mm, a size of 0.27 mm and oriented in the direction of blood flow at an angle of 80°. The maximum blood velocity measured over time was automatically adjusted to a sensitivity of x6. Maximum blood velocity (in mm.s^−1^) was measured in diastole and systole over 5 consecutive cycles, and averaged manually.

### Measurement of hemodynamic viscosity and hematocrit

Hemodynamic viscosity was determined on blood samples using a newly developed setup and methodology (Hemovis, Fontenay-sous-Bois, France). A situation similar to the *in vivo* red blood cells and plasma plug flow separation effect is replicated in the device developed by Hemovis (de Tilly et al., 2023) allowing whole blood and plasma viscosity measurements and the plasma thickness estimation. Briefly, whole blood viscosity ( $\eta_{b}$ ) was measured, for each mouse, on 800 µL of blood at 3 kPa pressure in a 380 µm diameter tube (shear rate = 1,200 s^−1^) using Hemovis device. Then, all same sex mice blood samples were pooled and centrifugated at 2,000 g for 10 min to separate the plasma and the cellular elements of blood. Plasma viscosity ( $\eta_{p}$ ) was measured, for same sex mice pooled blood, using Hemovis device.

Hematocrit ( Ht ) was measured, for each mouse, on 50 µL of blood using scil Vet abc Plus + device (Scil Animal Care Company, Altorf, France).

### Calculation of carotid wall shear stress

**FIGURE 1:**
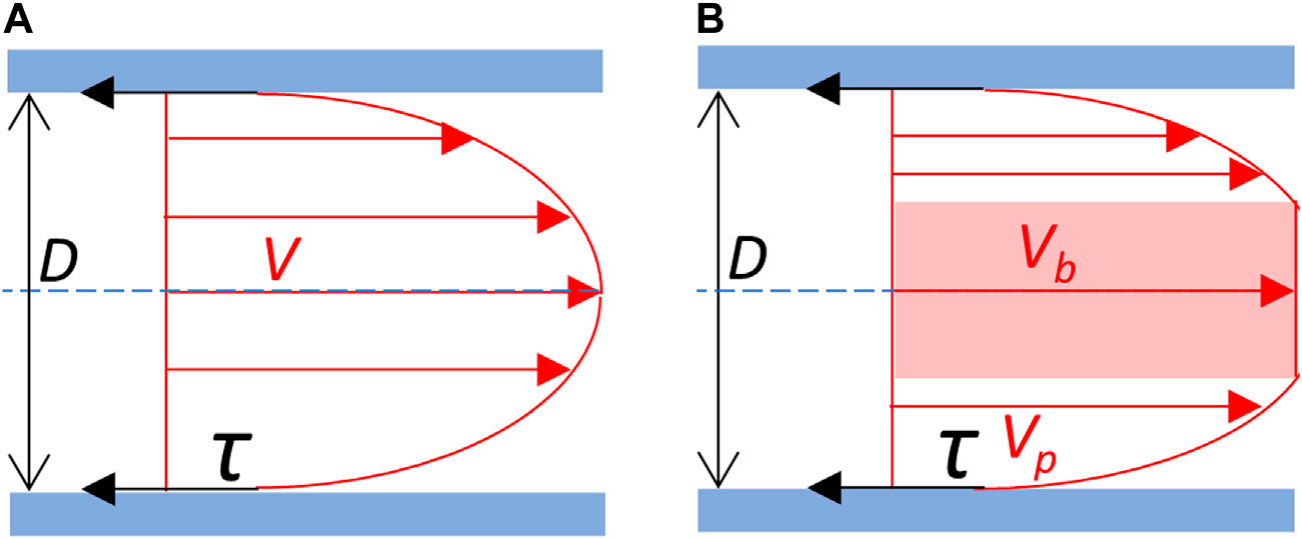
Blood flow velocity profile and wall shear stress. **(A)** Newtonian schematic representation of the velocity profile of the blood flow. Blood is considered as an homogeneous fluid which velocity profile (V) follows a parabolic curve, the maximal velocity being at the center of the vessel. **(B)** Schematic representation of the separation between red blood cells plug flow and plasma sheath flow on the velocity profile of the blood flow. Blood is considered as non-homogeneous. Cellular elements of the blood constitute a central blood plug (in pink), which velocity (Vb) corresponds to the maximal velocity, in fact the velocity of the plug flow. The plasma forms a peripheral sheath which velocity profile (*V*_*p*_) depends on its thickness. Red arrows: velocity (V). τ (black arrows): shear stress exerted on the vessel wall (in blue). D: vessel diameter. Blue dot line: central axis of the vessel.

The experimental data, i.e., carotid inner diameter, maximal blood flow velocity and parameters related to blood viscosity (total blood viscosity, plasmatic viscosity and hematocrit), have been used to calculate the carotid diastolic and systolic WSS using 2 different methods, the classical one, assuming Poiseuille’s law Newtonian fluid properties of the blood, and our own method based on the “Fahraeus-type” separation between red blood cells plug flow and plasma sheath flow, called in this article N-method and F-method, respectively (Figure 1). In both cases, WSS calculation was grounded on the basic principle according to which the WSS ( τ ) is the product of the viscosity η and the shear rate ( σ ) (Roux et al., 2020):

τ=η×σ (1)

The shear rate depends on the geometry of the vessel segment and the characteristics of the fluid flow, and the 2 methods differed (i) in the value chosen for η and (ii) in the way σ was calculated.

Calculation of WSS ( τ ) according to the N-method was based on the general equation:

$$\tau =\eta \times \frac{8\times V_{m}}{D}$$ (2)

where η is the blood viscosity, $V_{m}$ is the mean blood velocity, and D the vessel inner diameter. Since Doppler measurement provides not the mean velocity but the maximal one ( $V_{\max}$ ), WSS calculation should consider the relationship between $V_{m}$ and $V_{\max}$ , determined by the profile of blood velocity from the centre of the vessel, where the velocity in maximal, to the edges of the vessel. Under the assumption that blood behaves as a Newtonian fluid, the velocity profile is parabolic, and the ratio of $V_{\max}$ to $V_{m}$ is 2, so the WSS is given by the following equation:

$$\tau_{t}=\eta_{b}\times \frac{4\times V_{\max}}{D}$$ (3)

where $\tau_{t}$ is the theoretical WSS, $\eta_{b}$ is the total blood viscosity, $V_{\max}$ is the maximal blood velocity, and D the carotid inner diameter. Eq. 3 was applied to calculate the WSS according to the so-called “theoretical N-method,” using, for each animal, individual values of vessel diameter, maximal blood velocity, and blood viscosity.

However, it has been shown that in small arteries, the $V_{\max}$ to $V_{m}$ ratio is usually between 1.39 and 1.54, i.e., less than the theoretical value 2 (Reneman et al., 2006), and the apparent blood viscosity is lower than in larger vessels. These differences from the theoretical values are due to the fact that blood cells concentrate in the central core of blood flow, surrounded by a fluid peripheral sheath of plasma with some blood cells in suspension, known as the Fahraeus-Lindqvist effect, but this heterogenous blood behavior can be considered in an approximate way with a corrected Newtonian-based equation, as follows:

$$\tau_{c}=\eta \times \frac{5,5\times V_{\max}}{D}$$ (4)

where $\tau_{c}$ is the corrected WSS, η is the fluid viscosity, $V_{\max}$ is the maximal blood velocity, and D the carotid inner diameter, assuming a $V_{\max}/V_{m}$ ratio equal to 1.45. Eq. 4 has been applied to calculate the WSS according to the so-called “corrected N-method,” using, for each animal, individual values of vessel diameter, maximal blood velocity, and 3 different values for η : the average plasmatic viscosity calculated on the pooled plasma of each animal group ( $\eta_{p}$ ), the individual total blood viscosity ( $\eta_{b}$ , and a apparent viscosity ( $\eta_{app.}$ ) obtained using a correcting factor for $\eta_{p}$ . In accordance with the literature, we set the correcting factor at 1.78 ( $\eta_{app.}=1.78\times \eta_{p}$ ) (Secomb, 2017).

By contrast with the N-method, calculation of WSS according to the F-method ( $\tau_{F}$ ) considers a non-homogenous behavior of the blood flow, with a central solid-like blood plug, constituted by the cellular elements of the blood, circled by a peripheral plasmatic sheath responsible for the actual WSS, which hence depends on the plasmatic viscosity and the shear rate ( $\sigma_{F}$ ):

$$\tau_{F}=\eta_{p}\times \sigma_{F}$$ (5)

As for $\sigma_{F}$ , it depends on (i) the velocity of the central blood plug, corresponding to $V_{\max}$ , (ii) the vessel diameter, (iii) the thickness of the plasmatic sheath, and (iv) the velocity profile within the plasmatic sheath. Based on a previous 2D model (de Tilly, 2015), applying the physical formulation of the blood plug and sheath flow separation model to the real vessel, we developed a 3D equation allowing to calculate $\sigma_{F}$ from the maximal blood velocity ( $V_{\max}$ ), the vessel radius ( R=D/2 ) and hematocrit (Ht) (see Supplementary Material):

$$\sigma_{F}=\frac{{2V}_{\max}}{R}\times \frac{\left(1-Ht\right)}{Ht\times \left(1-2\ln\sqrt{Ht}\right)-1}$$ (6)

The WSS can be calculated as follows:

$$\tau_{F}=\eta_{p}\times \frac{4V_{\max}}{D}\times \frac{\left(1-Ht\right)}{Ht\times \left(1-lnHt\right)-1}$$ (7)

In this equation, $\sigma_{F}$ is negative, the direction of the shear stress vector being opposite to that of the blood flow. To present the WSS as positive, as it is usual done, and to make easy comparison with the N-method equation, the absolute value of $\sigma_{F}$ was calculated from Eq. 7 as follows:

$$\tau_{F}\vee \eta_{p}\times \frac{{4V}_{\max}}{D}\times \frac{\left(Ht-1\right)}{Ht\times \left(1-lnHt\right)-1}$$ (8)

where $\eta_{p}$ is the blood plasma viscosity, $V_{\max}$ is the maximal blood velocity, D the carotid inner diameter and Ht the hematocrit. Eq. 8 has been applied to calculate the WSS according to the so-called “F-method,” using, for each animal, individual values of vessel diameter, maximal blood velocity and hematocrit, and the average plasmatic viscosity calculated on the pooled plasma of each animal group.

### Endothelial cells planar polarity and orientation

#### Carotid preparation

After euthanasia, a sternotomy was carried out to catheterize the left ventricle. A perfusion of physiological solution at 80 mm Hg pressure for 3 min was done to remove the blood from the vasculature. A second perfusion of 4% formalin (10% neutral buffered formalin, DiaPath, Martinengo, Italy) was done for 5 min to fix the tissues. The right common carotid artery was then carefully harvested with its bifurcation, to determine the direction of blood flow, and gently opened in two to allow better penetration of the immunostaining products. It was then placed in 4% formalin for 30 min at room temperature.

#### Carotid *in toto* immunostaining

To study the orientation and polarity of right common carotid artery ECs, Golgi apparatus immunostaining and nuclear labelling were performed on carotid arteries *in toto* in a 350 µL flat-bottom 96-well plate (Dutscher, Bernolsheim, France). For each bath or wash, the carotids were placed in 200 µL of the solution. The plate was placed on a shaker with horizontal agitation at 300 rpm.

Following the formalin fixation, the carotid arteries were washed twice with PBS1X for 7 min each at room temperature. Antigen retrieval was performed for 4 min at 95°C in an Eppendorf tube containing a solution of 1M Tris (TRIS Base Ultrapure, Euromedex, Souffelweyersheim, France)/0.5M EDTA (EDTA, Euromedex, Souffelweyersheim, France) at pH 8 to reveal the antigenic sites for the primary antibody. Three 5-min washes with PBS1X were performed. Non-specific binding sites were saturated for 1 h at room temperature using a saturation solution containing 10% donkey serum (Donkey Normal Serum (UP77719A K), Interchim Uptima, Montluçon, France), 0.5% Triton X-100 (Triton X-100 (T8532), Sigma-Aldrich, Missouri, United States), 0.01% Deoxycholic Acid Sodium Salt (BP 349-100), ThermoFisher Scientific, Massachusetts, United States) in PBS1X. Incubation with rabbit primary antibody anti-mouse Golgi integral membrane protein 4 [Recombinant Anti-GOLPH4/GPP130 antibody (EPR13439) (ab197595), abcam, Cambridge, United States] was carried out overnight at 4°C with horizontal agitation at 70 rpm. The following day, three 10-min washes with PBS1X were performed at room temperature. Incubation with Donkey secondary antibody anti-rabbit IgG coupled to a green-emitting fluorochrome (Donkey anti-Rabbit IgG (H + L) Highly Cross-Adsorbed Secondary Antibody, Alexa Fluor™ 488 (A21206), ThermoFisher Scientific, Massachusetts, United States) was performed for 2 h at room temperature. A final 10-min wash at room temperature was performed.

The right common carotid artery was mounted between glass slide and coverslip using a mounting medium containing DAPI (Fluoromount-GTM, with DAPI (00-4959-52), ThermoFisher Scientific, Massachusetts, United States), for nuclei labelling. The right common carotid artery was oriented so that the endothelium was in contact with the coverslip and the bifurcation was at the top of the slide. It was stored in an opaque box at room temperature for at least 2 days until the images were acquired.

#### Image acquisition

2D images of the nuclei and Golgi apparatus of the endothelium of the right common carotid artery were acquired by confocal microscopy using an LSM 900 system (Jena, Germany) equipped an Axiocam 305 mono camera and a 40×/1 Plan-Apochromat objective. The tissue was illuminated consecutively by a with 405 nm laser to observe nuclei, and a 488 nm laser to observe Golgi apparatus. The pinhole was set at 3.03 Airy for the 405 nm laser and 2.81 Airy for the 488 nm laser, allowing acquisition of a section 1.5 µm thick. The laser power was set at 1.5% of the maximum power, and the gain at 600. The fluorescence wavelength emitted was collected at 452 nm to image the nuclei, and 520 nm for the Golgi apparatus. Imaging is performed by sampling an area measuring 160 × 160 µm. The spatial resolution of the system is 156 nm in (x, y). The final pixel size is 78 nm in (x, y). At the end of acquisition, the images obtained are in 8-bit format.

#### Image processing

Image processing was performed using the open-source ImageJ/Fiji software [ImageJ 2.1.0/1.53h/Java 1.8.0_66 (64 bit)]. Segmentation was used to obtain binarized images of the nuclei and Golgi apparatus. A threshold, based on the Gaussian distribution of the background voxel grey-level histogram technique, was calculated for each image by adapting previously developed thresholding technique for vascular network (Nicolas et al., 2023). Threshold was set at the mean + 3.89xSD to eliminate the background and 99.995% of the unlabeled vascular tissue voxel population. After binarization of the segmented images, a median filter set at 3 voxels for (x, y) was applied to suppress artefactual isolated voxels. After segmentation and filtering of the Golgi apparatus and nuclei images, the two images were merged into a single RGB image, with the nuclei in blue and the Golgi apparatus in green. After merging the images, some artefacts remain, corresponding to small blue and green objects that are neither nuclei nor Golgi apparatus. To identify only the nuclei and Golgi apparatus, two successive segmentations based on the color and size of the objects were carried out. Segmentation based on color identified all objects of the same color. Nuclei and associated artefacts were thresheld on blue, and Golgi apparatus and associated artefacts were thresheld on green. Size segmentation was used to exclude small artefacts that corresponded neither to nuclei nor to Golgi apparatus, so that only nuclei and Golgi apparatus could be identified and numbered. The thresholds for identifying nuclei and Golgi apparatus by size are 10 μm^2^ and 2 μm^2^ respectively.

#### Data analysis

At the end of the nuclei and Golgi apparatus identification stage, a unique ID number was obtained for each object (nucleus and Golgi apparatus), with the orthonormal coordinates of its center in (x,y), and the length (in µm) of its major and minor axes. These data were processed, using Microsoft Excel with the Kutools extension, to calculate the orientation and the polarity of ECs as follows.

For each EC orientation, the “nucleus-Golgi apparatus” (N-G) vector was first identified by the coordinates of its starting point (center of the nucleus) and its ending point (center of the nearest Golgi apparatus). The angle between each N-G vector and the direction of the blood flow was then calculated (in degree, reported on a semicircle) and used to define the orientation of each EC from blood flow, 0° and 180° being the orientations similar and opposite to the blood flow, respectively. Three classes of orientation were then defined according to the N-G vector angle: (i) a dromic orientation (in the direction of the flow) when the angle range was [0°; 60°]; (ii) a perpendicular orientation (to the direction of the flow) when it was [60°; 120°]; (iii) an antidromic orientation (in the opposite direction to the flow) when it was [120°; 180°]. The frequency of angle distribution was studied for each carotid. In the case of an equiprobable distribution, corresponding to the absence of orientation, the angle frequency is one-third per class.

The polarity of the ECs of the right common carotid artery was defined by two parameters, the length (in µm) of the N-G vector and the elongation coefficient of the nucleus corresponding to the ratio between the lengths of the major and minor axes of the nucleus.

### Statistical analysis

All statistical analyses were done using GraphPad Prism^®^ software (Prism 9.0.1 version), (San Diego, CA, United States), and results were considered as statistically significant for *p* < 0.05. Hematocrit, viscosity, and shear stress parameters are expressed as mean ± standard deviation (SD). Statistical comparisons between male and female were done with Mann-Whitney non-parametric test. Hematocrit versus blood viscosity was analyzed by linear regression. Cell orientation parameters were analyzed using a contingency table with three outcomes, and compared with χ^2^ test. Polarity parameters were expressed as mean ± SEM. Statistical comparisons were done with one-sample Wilcoxon signed-rank test (nucleus elongation index) and Mann-Whitney non-parametric test (male versus female).

## Results

### Blood flow velocity and vessel diameter

**FIGURE 2:**
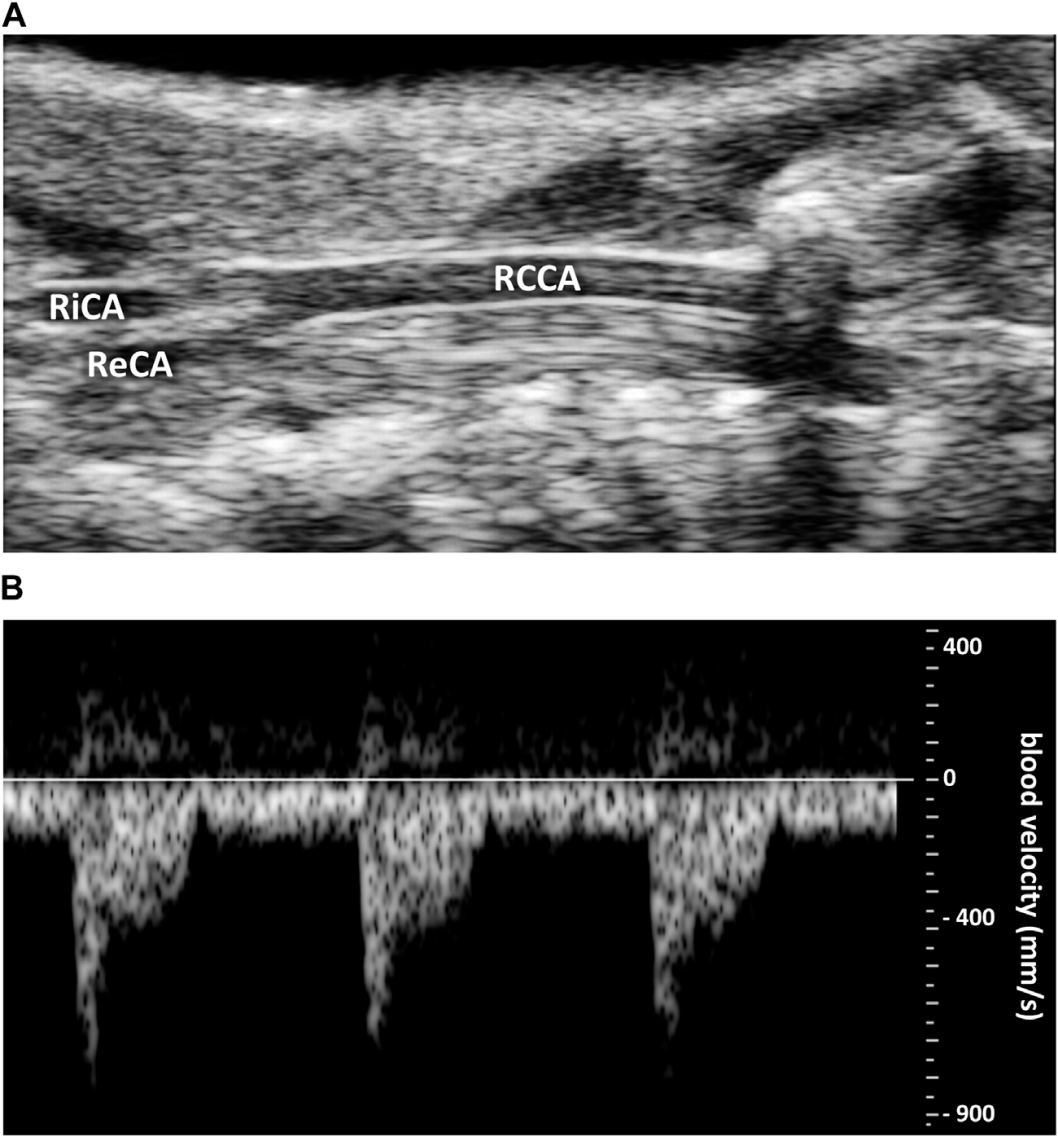
Representative original traces of carotid ultrasound imaging. **(A)** Right common coronary artery (RCCA) diameter. RiCA, right internal carotid artery. ReCA, right external carotid artery. **(B)** Blood velocity profile (in mm.s^−1^).

**FIGURE 3:**
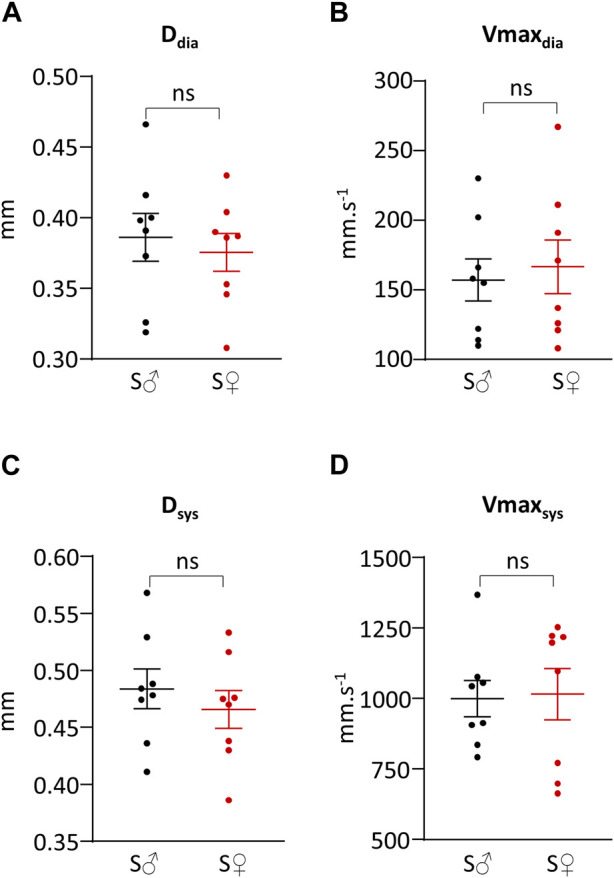
Systolic and diastolic right common carotid artery diameter and blood velocity. **(A)** Vessel diameter during cardiac diastole (D_dia_) in mm. **(B)** Maximum blood velocity during cardiac diastole (Vmax_dia_) in mm.s^−1^. **(C)** Vessel diameter during cardiac systole (D_sys_) in mm. **(D)** Maximum blood velocity during cardiac systole (Vmax_sys_) in mm.s^−1^. Measurements were done on 8 C57BL6/J male mice (S♂), and on 8 C57BL6/J female mice (S♀). Dots are individual values. Horizontal bars are means, and vertical bars car SEM. Data were compared by Mann-Whitney test. n.s. = non significant.

Representative original traces of right common carotid artery diameter and blood flow velocity measurements are shown in Figure 2. Systolic and diastolic arterial diameters and maximum blood velocities are given in Table 1 and presented in Figure 3. For all parameters, differences between male and female were very low, less than 6%, and were not statistically significant. Average mean diastolic diameter was around 0.380 mm, with around 25% increase in systole. Average mean diastolic maximum blood velocity was around 160 mm.s^−1^, with more than 500% increase in systole.

**TABLE 1:** Hemodynamic parameters. Values are given as mean ± standard deviation (SD), for 8-week old C57BL6/J male (S♂) and female (S♀) mice. bpm: beats per minute; WSS: wall shear stress. Shear stress data in italics are calculated from the experimental data using (Eq. 3) for theoretical N-method (Eq. 4), for corrected N-method, with apparent viscosity = 2.24 (♂) and 2.26 (♀), applying 1.78 as correcting factor to plasmatic viscosity, and (Eq. 8) for F-method.

	S♂ (n = 6–8)		S♀ (n = 7–8)	
Hematocrit	0.43 ± 0.02		0.43 ± 0.03	
Total blood viscosity (cP)	4.1 ± 0.4		3.9 ± 0.6	
Plasma viscosity (cP)	1.26		1.27	
Heart frequency (bpm)	455 ± 36		429 ± 62	
	Diastole	Systole	Diastole	Systole
Diameter (mm)	0.387 ± 0.05	0.484 ± 0.05	0.376 ± 0.04	0.466 ± 0.05
Blood velocity (mm.s^−1^)	157 ± 43	999 ± 183	167 ± 54	1,015 ± 258
Theoretical N-method WSS (Pa)	*6.1 ± 1.5*	*34.7 ± 8.1*	*6.9 ± 2.9*	*33.4 ± 9.4*
Corrected N-method WSS (Pa)	*5.2 ± 1.9*	*25.9 ± 5.9*	*5.6 ± 2.3*	*27.2 ± 7.0*
F-method WSS (Pa)	*5.9 ± 2.3*	*29.7 ± 6.5*	*6.3 ± 2.5*	*30.4 ± 6.9*

### Hemodynamic viscosity and hematocrit

**FIGURE 4:**
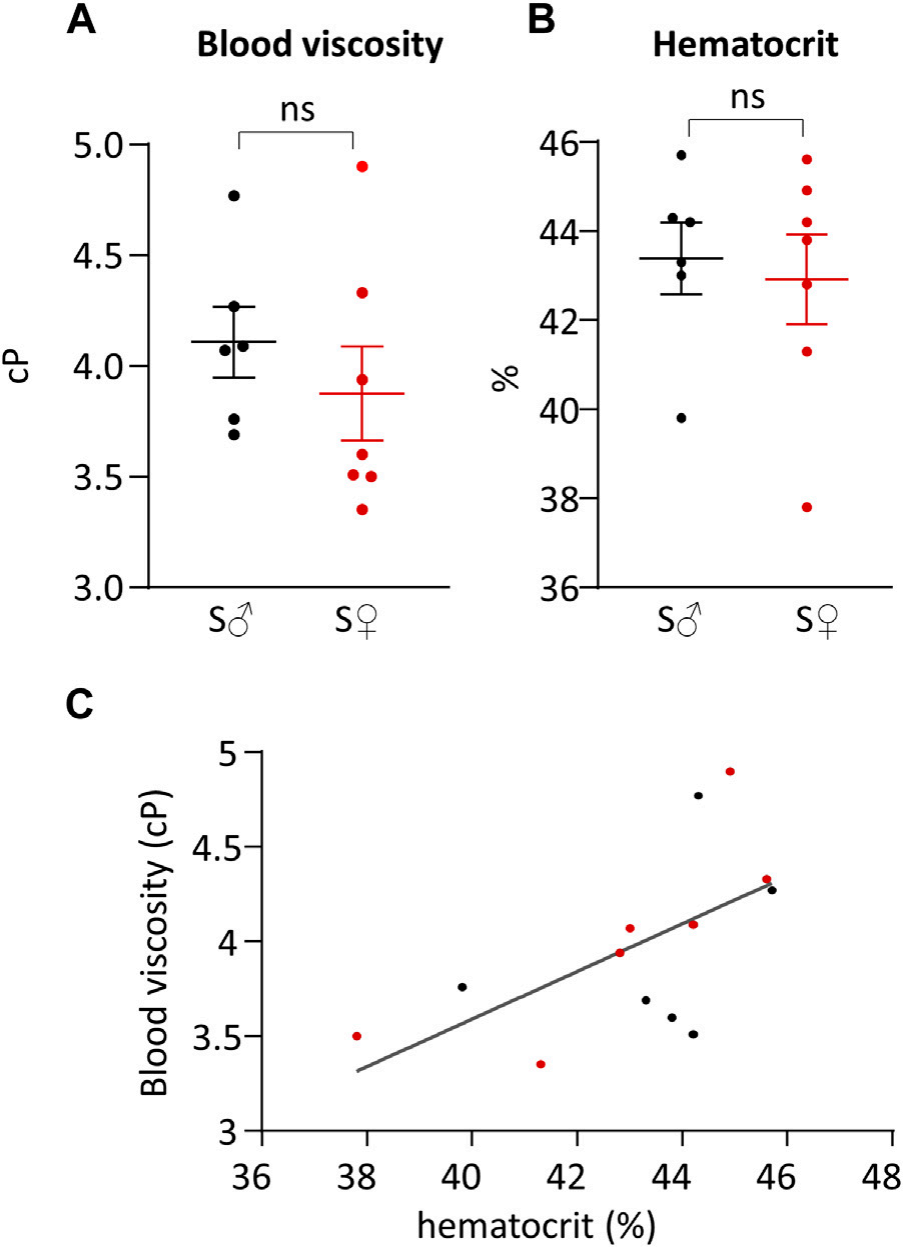
Total blood viscosity and hematocrit. **(A)** Total blood viscosity in cP. **(B)** Hematocrit in %. Measurements were done on 6 C57BL6/J male mice (S♂), and on 7 C57BL6/J female mice (S♀). Dots are individual values. Horizontal bars are means, and vertical bars car SEM. Data were compared by Mann-Whitney test. n.s. = non significant. **(C)** Total blood viscosity plotted against hematocrit. Data were fitted by linear regression. Black dots are male individual values, and red dots are female individual values.

Total blood viscosity and hematocrit are given in Table 1 and presented in Figures 4A, B, respectively. Differences between male and female blood viscosity were very low, equal to 5%, and were not statistically different. Individual hematocrit varied between 38% and 46%, without difference in mean value between male and female.

The relationship between total blood viscosity and hematocrit was evaluated by plotting blood viscosity ( $\eta_{b}$ ) against the hematocrit ( Ht ) (Figure 4C) with linear regression analysis, providing the following equation (R^2^ = 0.35):

$$\eta_{b}=\left(0.01258\times Ht\right)-0.001443$$ (9)

Plasma viscosity was measured, for each sex, on pooled blood of each sex group. Male plasma viscosity was equal to 1.26 cP, and female plasma viscosity was equal to 1.27 cP.

### Shear stress

**FIGURE 5:**
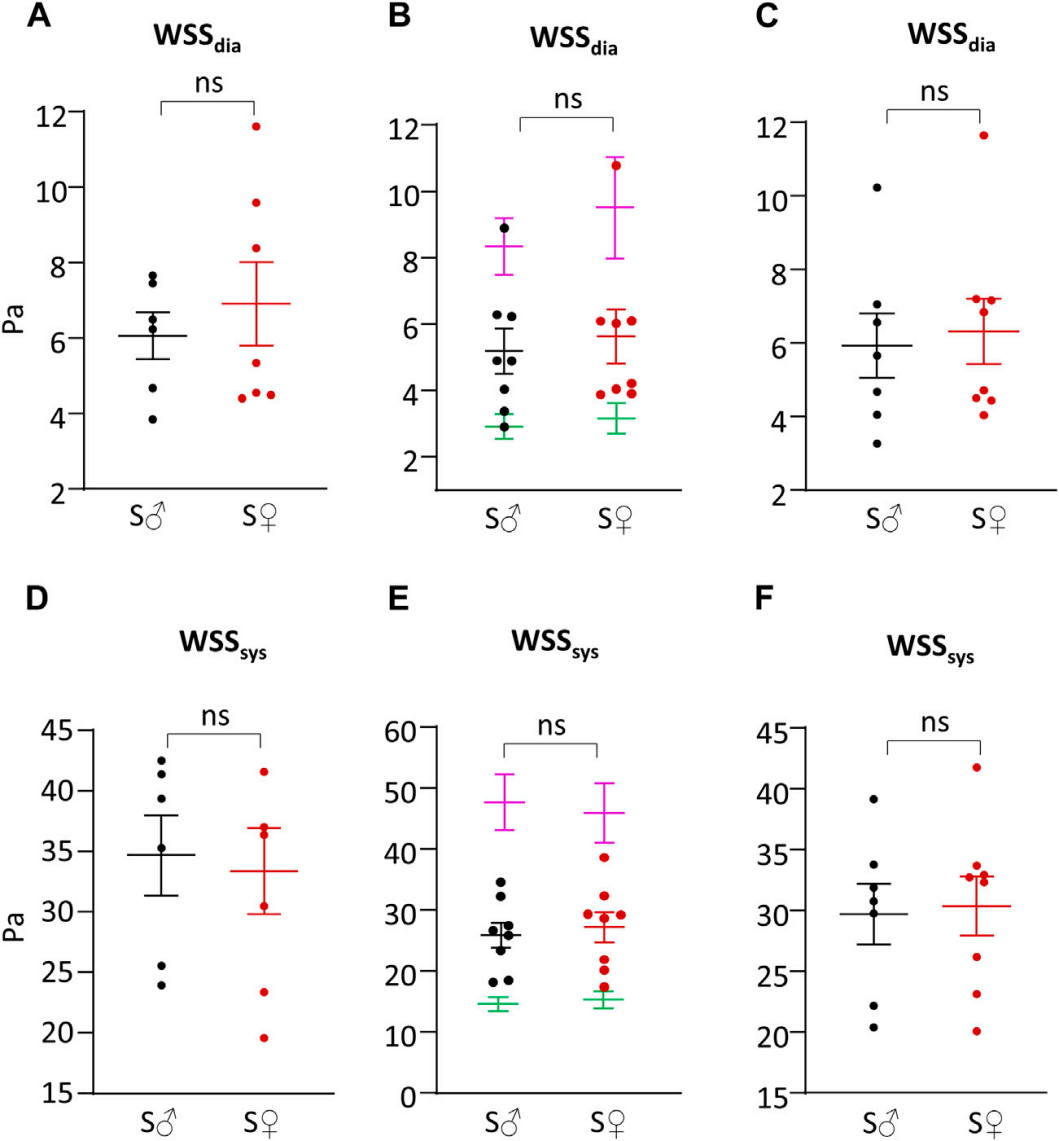
Shear stress. **(A)** Wall shear stress value during cardiac diastole (WSS_dia_) in Pa, calculated with theoretical N-method (Eq. 3). **(B)** Wall shear stress value during cardiac diastole (WSS_dia_) in Pa, calculated with corrected N-method with plasmatic (green), total blood (purple) and apparent (=1.78 plasmatic) (female, red/male, black) viscosity (Eq. 4). **(C)** Wall shear stress value during cardiac diastole (WSS_dia_) in Pa, calculated with F-method (Eq. 8). **(D)** Wall shear stress value during cardiac systole (WSS_sys_) in Pa, calculated with theoretical N-method (Eq. 3). **(E)** Wall shear stress value during cardiac systole (WSS_sys_) in Pa, calculated with corrected N-method with plasmatic (green), total blood (purple) and apparent (=1.78 plasmatic) (female, red/male, black) viscosity (Eq. 4). **(F)** Wall shear stress value during cardiac systole (WSS_sys_) in Pa, calculated with F-method (Eq. 8). Measurements were done on 6-8 C57BL6/J male mice (S♂), and on 7-8 C57BL6/J female mice (S♀). Dots are individual values. Horizontal bars are means, and vertical bars car SEM. Data were compared by Mann-Whitney test. n.s. = non significant.

Systolic and diastolic shear stress values were calculated by the theoretical N-method (tN-method), corrected N-method (cN-method), and F-method (Table 1; Figure 5). Regarding sex difference, whatever the calculation method used, differences between male and female mice for systolic and diastolic WSS values were very small, ranging from less than 1% to 13%, and were not statistically different.

cN-method generated identical WSR values but very different WSS ones depending on the viscosity value, as shown in Figures 5B, E, with a 3-fold difference between plasmatic and total blood viscosity, the value obtained with apparent viscosity (corrected from plasmatic viscosity) being in between. WSS values with plasmatic and total blood viscosity were very different from that obtained using the tN- and F-methods. By contrast, cN-method with corrected apparent viscosity provided WSS values close to that obtained from tN-method and F-method, themselves being very similar for both diastolic (6% difference) and systolic (14% difference) WSS values.

**FIGURE 6:**
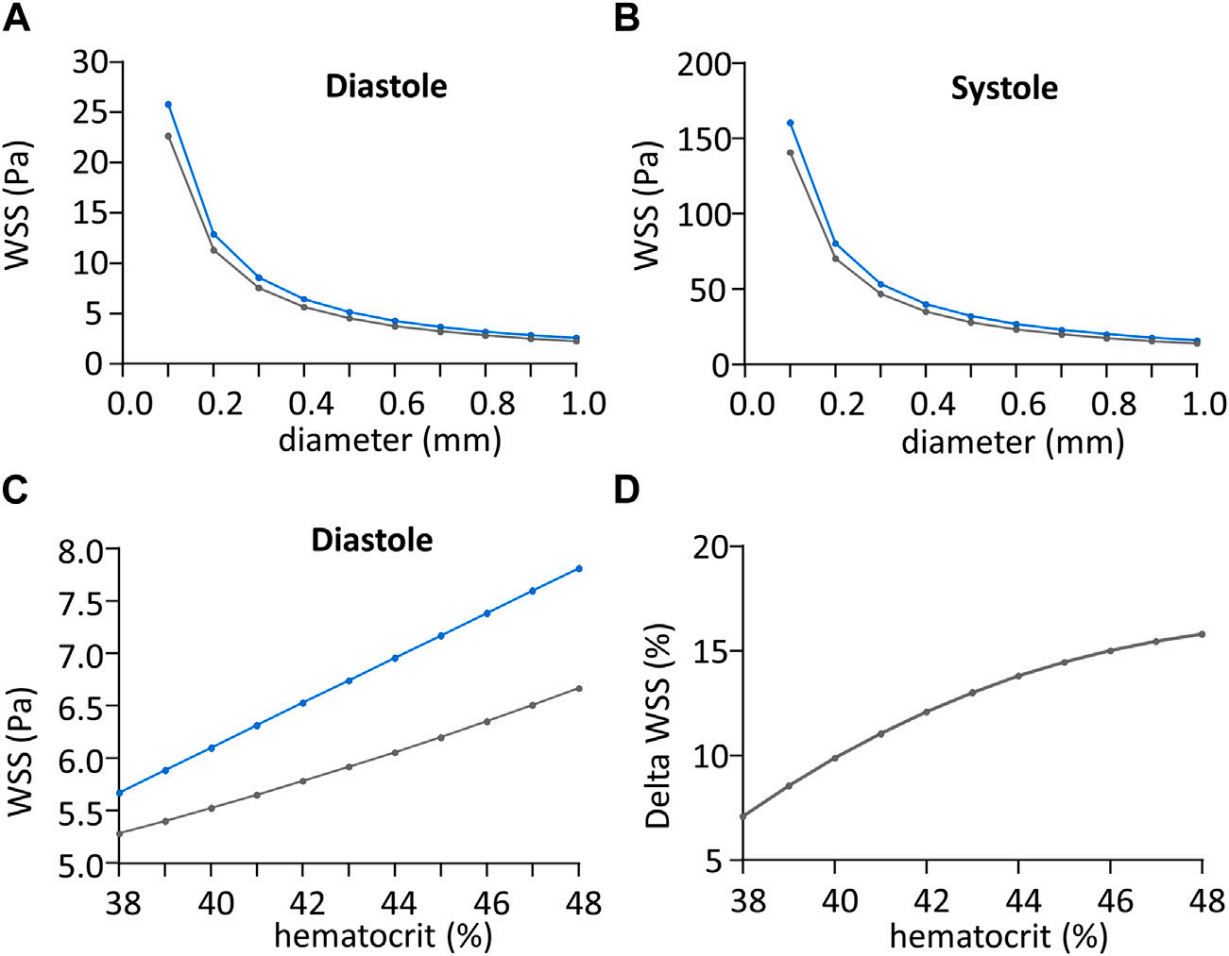
Impact of shear stress calculation method regarding vessel diameter. **(A)** Wall shear stress value (WSS) during cardiac diastole in Pa, regarding vessel diameter in mm, calculated with theoretical N-method (in blue) and F-method (in grey). **(B)** Wall shear stress (WSS) value during cardiac systole in Pa, regarding vessel diameter in mm, calculated with theoretical N-method (in blue) and F-method (in grey). **(C)** Wall shear stress (WSS) value during cardiac diastole in Pa, regarding hematocrit in %, calculated with theoretical N-method (in blue) and F-method (grey). **(D)** Difference between theoretical N-method and F-method wall shear stress (WSS) values normalized to the mean WSS from the two methods, in Pa, plotted against hematocrit in %.

Beyond the calculation of WSS with our own experimental results, we have used Eqs 3, 8 to perform a comparative sensitivity analysis of the tN-method and F-methods to vessel diameter, blood flow velocity, and hematocrit. For this, we generated WSS predictions of the tN-method and F-methods changing each of these parameters, the others being set constant. Since no sex difference was seen, data from each sex were pooled for these comparisons. Since the Fahraeus-Lindqvist effect is usually considered to occur in small vessels (diameter <0.3 mm), we have first analyzed the relationship between vessel diameter ranging from 0.1 to 1 mm (with 0.1 mm step) and WSS predicted by the tN- and F-methods, respectively, for our average diastolic and systolic $V_{\max}$ . Whatever the diameter, the difference between tN- and F-methods was 13.03% (Figures 6A, B). A similar approach was used to analyze the relationship between blood maximal velocity (ranging from 100 to 1,100 mm.s^−1^) for our average diastolic and systolic diameters, with identical results (13.03% difference) whatever the blood maximal velocity (data not shown). For the analysis of hematocrit variation, since this parameter is not formally present in the tN-method, we have implemented Eq. 3 with the linear Eq. 9 obtained from the regression analysis of the hematocrit-viscosity analysis, as follows:

$$\tau_{t}=\left(\left(0.01258\times Ht\right)-0.001443\right)\times \frac{4\times V_{\max}}{D}$$ (10)

The relationship between hematocrit, ranging from 38% to 48% mm (the range observed in our own experiments) and WSS predicted by the tN- and F-methods, respectively, calculated for our average diastolic $V_{\max}$ and D , is shown in Figure 6C. The difference in the predicted values by the 2 methods depended on the Ht value, the difference increasing with increasing hematocrit, ranging from 7% to 16% for hematocrit ranging from 38% to 48% (Figure 6D). Identical results were obtained for average systolic $V_{\max}$ and D (data not shown).

### EC polarity and orientation

**FIGURE 7:**
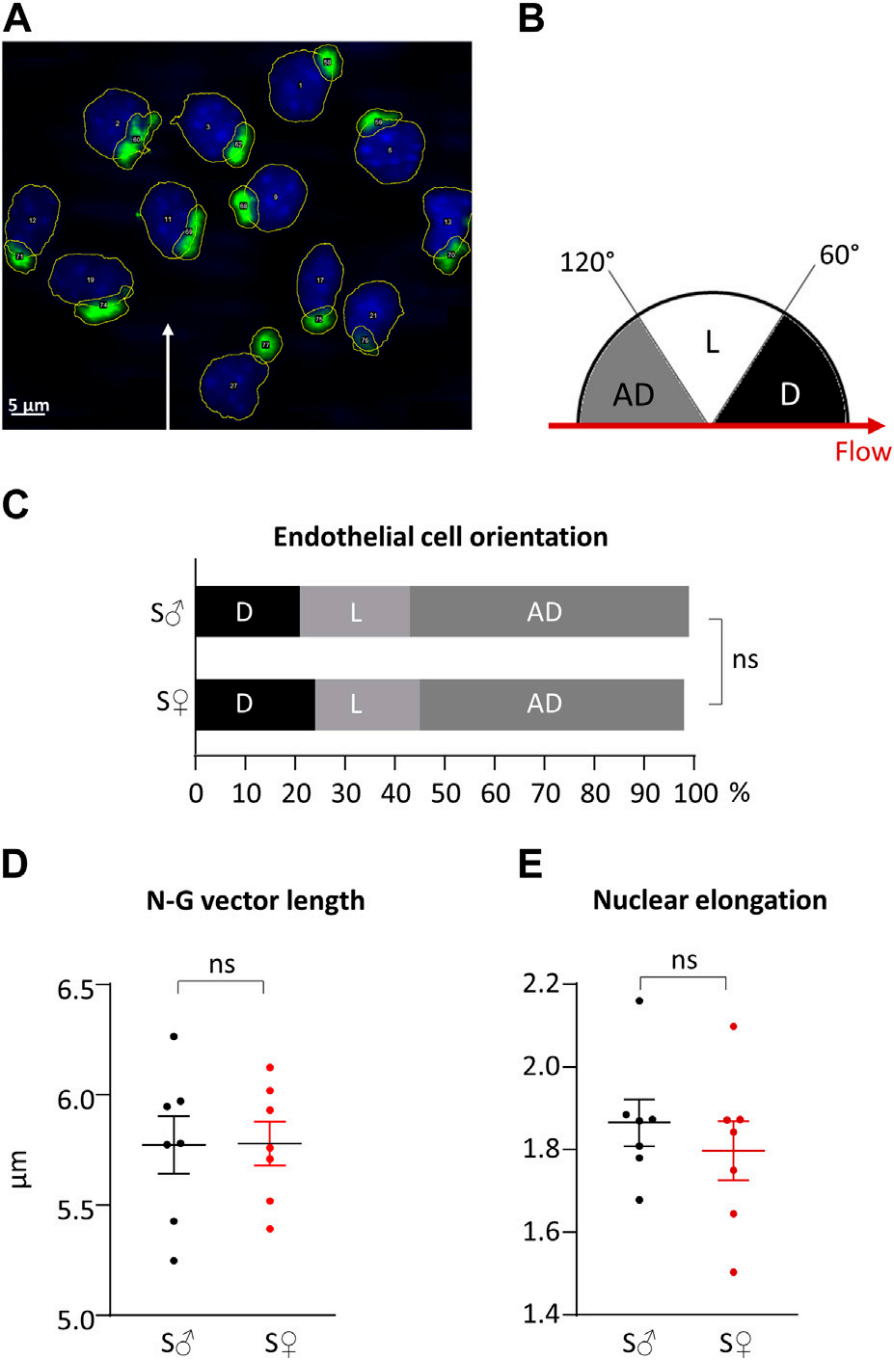
Right common carotid artery endothelial cells orientation and polarity. **(A)** Representative histological labeling and automated identification of nuclei and Golgi apparatus. Nuclei are in blue. Golgi apparatus are in green. Automatic contouring are drawn in yellow. Automatic identification and numbering are in written in white in the center of the organelle. White arrow represents blood flow direction. **(B)** Classification of cell orientations depending on blood flow direction. Three classes of cell orientation (in degree, on a semicircle) were defined based on the angle between the nucleus-Golgi apparatus vector and the direction of the blood flow. “AD”: antidromic (in grey); “D”: dromic (in black); “L”: lateral (in white). **(C)** Endothelial cells orientation in cell %. Measurements were done on 231 endothelial cells from 7 C57BL6/J male mice (S♂), and 335 endothelial cells from 7 C57BL6/J female mice (S♀). Data were compared using $\chi^{2}$ test. n.s. = non significant. **(D)** Nucleus-Golgi apparatus (N-G) vector length in µm. **(E)** Nucleus elongation. Measurements were done on 7 C57BL6/J male mice (S♂), and on 7 C57BL6/J female mice (S♀). Dots are individual values. Horizontal bars are means, and vertical bars car SEM. Data were compared by Mann-Whitney test. n.s. = non significant.

A representative image of nucleus and Golgi apparatus automated identification is shown in Figure 7A. The percentage of antidromic, dromic and lateral EC orientation (angle between N-G vector and blood blow direction, see Figure 7B) is presented in Figure 7C. For both sex, ECs were mainly oriented against blood flow, with more than 50% of antidromic ECs (statistically different from random orientation). Differences between male and female ECs orientation were very low, inferior to 15%, and were not statistically different.

Regarding EC polarization, nuclei were elongated with a nucleus elongation ratio statistically different from 1, for both sexes. EC polarization was similar in both sexes, since differences between male and female for the N-G vector length and nucleus elongation ratio were very low, less than 4%, and not statistically different. The length of the N-G vector and the elongation of the nuclei are presented in in Figures 7D, E, respectively.

## Discussion

Our results showed that mouse common coronary artery diameter varies between 350 µm and 550 μm, approximatively, between cardiac diastole and systole. While being more than ten-fold thinner than average human carotid artery diameter (Roux et al., 2020), our results are in accordance with mouse literature values (Panteleev et al., 2021). Regarding the maximum blood velocity, our values vary between 150 mm.s^−1^ (diastole) and 1,000 mm.s^−1^ (systole). Compared to literature on mouse carotid artery that shows a variation between 250 mm.s^−1^ and 1,800 mm.s^−1^ (Parzy et al., 2009), our own values are a little bit lower. However, this difference can be correlated to the heart beat effect, as it can be seen that the higher the heart beats are, the higher the blood velocity values are too, and vice versa (Chérin et al., 2006; Parzy et al., 2009).

Our results showed that mouse average hematocrit is around 43%, which is in accordance with average values found in literature around 40%–42% (Vogel et al., 2003; Windberger et al., 2003). While it has been already shown for humans that hematocrit values are higher for man compared to woman (Grau et al., 2018), to our study showed that there were no difference between male and female hematocrit in mice. Average total blood viscosity in our study was found around 4 cP. It has been shown that mouse total blood apparent viscosity decreases from 30 cP to 4 cP when the shear rate value increases from 0 s^−1^ to 1,000 s^−1^, following an exponential decay (Vogel et al., 2003; Windberger et al., 2003; Alexy et al., 2022). As we measured this apparent blood viscosity at 1,200 s^−1^, our average value is in accordance with literature observation. Our results also confirmed previous observation regarding the linear relationship between the total blood viscosity and the hematocrit (Vogel et al., 2003). Our values for mouse plasma viscosity are in accordance with literature values (Vogel et al., 2003; Windberger et al., 2003).

WSS calculations were done under 2 different and opposite assumptions regarding the rheological properties of blood flow, both being idealized representations of actual blood flow. Importantly, they produced similar results, the differences between methods being small and not statistically significant, suggesting that these methods are similarly relevant.

In the so-called “Newtonian” method, blood is considered as a homogenous fluid following Poiseuille’s law Newtonian fluid properties, whereas the so-called « Fahraeus-Lindqvist » method considers that the blood flow is heterogeneous, with a central plug of blood cells separated from the plasma sheath, equivalent to the phenomenon initially described by Fahraeus in small tubes, and known as the “Fahraeus-Lindqvist effect.” It is generally accepted that this effect occurs in vessels of less than 300 µm diameter, close but inferior to the inner diastolic and systolic diameters of the mouse common carotid. It could be hence hypothesized that blood flow had an intermediate behavior in our model, the reason why we tested an empirical adaptation of the theoretical « Newtonian » approach, the so-called « corrected » N-method. Though opposite in their theoretical assumptions and different in their mathematical formulations, the theoretical N- and F-methods provided very similar results, that may be explained by the fact that the measured total blood viscosity, used for tN-method, is an apparent one assuming that blood is a homogenous fluid. This was also true for the corrected N-method when a correcting factor was applied to the plasmatic velocity to obtain the apparent viscosity. By contrast, the corrected N-method results with uncorrected blood viscosity, either plasmatic or total blood ones, were very different, being much lower and much higher, respectively, then that obtained with the other methods. Though it is known that the peripheral sheath directly in contact with the vessel wall is cell-free plasma (Secomb and Pries, 2013), taking into account the plasmatic viscosity produced irrelevant results. This is due to the fact that correction of the N-method, supposedly more realistic, remains an idealized representation of the blood as a Newtonian fluid and hence requires to rectify the plasmatic viscosity using a correcting factor. The key question is hence the choice of the correcting factor, in particular because the corrected N-method is highly sensitive to the viscosity value, and the correcting factor is not directly obtained from our experimental results. Additionally, the velocity profile could not be experimentally determined due to technical limitation, so that the $V_{\max}/V_{m}$ ratio was also extrapolated from studies done in conditions different from that of the present study. Though supposedly being realistic, taking into account the experimental data obtained in our study, this kind of approach is not really realistic, since it is highly sensitive to parameters that cannot be experimentally determined. By contrast, the F-method proposed in this study does not require such corrections and allows the calculation of shear rate and shear stress using non corrected experimental values for the different parameters of the corresponding equation. It is based on an idealization of the blood behavior, considering that all the blood cells constitutes the central core, and the peripheral sheath as cell-free plasma, whereas blood cells are actually present in suspension in the plasma sheath, except its peripheral layer directly in contact with the vessel wall (Secomb, 2017). However, comparison with *in vitro* measurement of blood flow (Yeleswarapu et al., 1998) shows that the theoretical velocity profile hypothesized in the F-method is realistically relevant.

Regarding the theoretical N- versus the F-method, the relative difference in their predictions remains identical under variation of the vessel diameter below and above the « threshold » value under which the Fahraeus-Lindqvist effect is supposed to occur. This may be surprising considering the actual blood flow behavior, but is mathematically logical since for both methods, the mathematical dependence of WSS on vessel diameter is identical, as it is for the maximal velocity (see Eqs 3, 8). This is not the case for the effect of the hematocrit on WSS value, since hematocrit is a parameter of the F-method but not the N-one. As discussed above, the total blood viscosity is indeed dependent on the hematocrit, but the empirical correlation is weak and hence of poor predictive value. Moreover, the total blood viscosity needed for the calculation of the WSS with the tN-method is an apparent one which value depends not only on the hematocrit but also the shear rate, which is not the case for the plasmatic viscosity (Vogel et al., 2003). Since the shear rate varies between diastole and systole, unbiased calculation of WSS using the total blood viscosity should consider different viscosity values for diastolic and systolic WSS, which is not the case for the F-method that used the plasmatic viscosity. Whatever the method used, our results showed highly oscillatory SS values during the cardiac cycle. Although these values are around 10-fold higher than those measured in the human common carotid artery (Roux et al., 2020), they are consistent with those found in the literature in mice (De Wilde et al., 2016).

Our results showed that mouse ECs were polarized and mainly oriented against the blood flow. This is in accordance with previous studies that showed that, physiologically, ECs are elongated (Urschel et al., 2021) and oriented against the blood flow (Franco et al., 2015; Vion et al., 2021). According to the literature, these characteristics of EC planar cell polarity are related to the high wall shear stress. ECs exposed to low WSS tend to show a “cobble-stone like shape” (Urschel et al., 2021), and more likely oriented with the blood flow direction (Baeyens et al., 2015; Vion et al., 2021).

In conclusion, our study provides, for both male and female mice, hemodynamic values for blood flow velocity and vessel diameter, combined with *in vitro* hematocrit and viscosity measurements on each mouse, allowing individual calculation of WSS variation during the cardiac cycle. We propose a new method for WSS calculation, alternative to the classical one based on the Poiseuille’s law, considering the so-called “Fahraeus-Lindqvist effect.” Comparison with experimental characterization of blood flow behavior indicates that this methodology is relevant to compute WSS in the macrovasculature, i.e., for vessel diameter above 100 µm. For small vessels, with diameter around 10 µm or lower, characterization of blood flow properties at microscale level requires further investigation. Additionally, regarding the potential influence of sex on WSS and EC sensitivity to blood flow direction, a comparison that, to the best of our knowledge, it has not been yet done in previous studies, it showed that WSS homeostasis was similar in male and female mice.

## Data Availability

The original contributions presented in the study are included in the article/Supplementary Material, further inquiries can be directed to the corresponding author.
