# Supplementary material for: Blood shear stress during the cardiac cycle and endothelial cell orientation and polarity in the carotid artery of male and female mice
Source: Front Physiol. 2024 Jul 12;15:1386151. doi: 10.3389/fphys.2024.1386151 (PMC11272658; doi:10.3389/fphys.2024.1386151)
Supplement: Supplementary file 1 [file DataSheet1.docx]

**Blood plug and sheath flow separation model: application to vessel wall shear stress calculation**

***Model of the sheath/plug flow***

In this model, red cells and plasma are separated. The blood cells constitute a central blood plug surrounded by a peripheral plasma sheath. The plug flow holds a constant mean velocity. The sheath flow holds a parabolic velocity profile (see. Fig.1B).

In the following equations,$r$ is the radial cylindrical coordinate, $R$ is the radius of the vessel, $b$ is the radius of the plug flow, $\delta=R-b$ is the thickness of the sheath flow. All the flow variables are equal on fixed $r$ by rotation.

$V_{max}$ is the velocity of the plug flow. Velocity at the wall $V(R)$ is null.

The flat velocity profile of the plug flow and the parabola in the sheath flow imply a velocity derivative be null on , $R=b$.

$V\left( b \right)=Vmax$ $V(R)=0$ ${(\frac{\partial V(r)}{\partial r})}_{r=b}=0$

***Velocity profile*** $V(r)$, ***wall shear rate*** ($\sigma_{w}$) and ***Velocity profile*** $V(r)$ ***and wall shear stress*** ($\tau_{w}$)

*B*y integrating the fluid equation in the sheath flow (Navier Stokes) $r\frac{\partial P}{\partial z}=\partial\frac{(\eta r(\frac{\partial v}{\partial r}))}{\partial r}$ ,

$V(r)=\alpha r^{2}+\beta\ln(\frac{r}{R})+\gamma$, where $\alpha$, $\beta$ and $\gamma$ are integration constants.

$V_{max}= \alpha b^{2}+\beta\ln\left( \frac{b}{R} \right)+\gamma$ ; $\alpha R^{2}+\gamma=0$ ; $(2\times\alpha\times R)+\beta/b=0$

$\alpha=\frac{V_{max}}{b^{2}-R^{2}-2b^{2}\ln(b/R)}$ ; $\beta=-2b^{2}\alpha$ ; $\gamma=-R^{2}\alpha$

$V(r)=\alpha(r^{2}-2b^{2}\ln(\frac{r}{R})-R^{2})$ ($\alpha$ is negative because $b<R$ : $\alpha<0$)

The wall shear rate $\sigma_{w} (r=R)$ is given by the following equation:

$$\sigma_{w}={(\frac{\partial V(r)}{\partial r})}_{r=R}=2\alpha\frac{R^{2}-b^{2}}{R}=\frac{2V_{max}}{b^{2}-R^{2}-2b^{2}\ln(b/R)}\times\frac{R^{2}-b^{2}}{R}$$

Since $\tau_{w}=\eta_{p}\times\sigma_{w}$, $\eta_{p}$ being the plasma viscosity,

$\tau_{w}=\eta_{p}2\alpha\frac{R^{2}-b^{2}}{R}$=$\eta_{p}\times\frac{2V_{max}}{b^{2}-R^{2}-2b^{2}\ln(b/R)}\times\frac{R^{2}-b^{2}}{R}$

***Wall shear stress*** ($\tau_{w}$) ***calculation from experimental data***

Ultrasound imaging provides the maximal velocity, i.e., $V_{max}$, the velocity of the plug flow, and the inner diameter $D=2R$ of the vessel. Blood analysis provides the plasmatic viscosity $\eta_{p}$ and the hematocrit $Ht$. In case of perfect separation between red cells and plasma, $Ht={b^{2}}/{R^{2}}$ and so $b^{2}=HtR^{2}$ and $b=R\sqrt{Ht}$.

Hence $\tau_{w}$ can be calculated from the experimental data obtained from ultrasound imaging and blood analysis as follows :

$\tau_{w}=\eta_{p}\times\frac{2V_{max}}{HtR^{2}-R^{2}-2HtR^{2}\ln(({R\sqrt{Ht})}/R)}\times\frac{R^{2}-HtR^{2}}{R}$

which, by simplification, leads to the following equation, corresponding to eq. (7):

$\tau_{w}=\eta_{p}\times\frac{{4V}_{max}}{D}\times\frac{(1-Ht)}{Ht\text{×}(1-lnHt)-1}$
